# Amblyopia Due to Dental Irritation

**Published:** 1893-05

**Authors:** 


### Amblyopia Due to Dental Irritation.

Dr. J. Walter Park reports (*Annals of Ophthalmology and Otology*) the following case, which came under his observation while at the Royal London Hospital:

The patient complained of headaches, blurred vision, etc. R. E. V.=6—36, L. E. V.=6—6, with correction of ametropia V=6—6 in either eye. However, she returned in a few days reporting rapid failure of vision of right eye; vision was reduced to counting fingers at ten inches. Action of pupil was sluggish; ophthalmoscopic examination revealed nothing to account for the trouble. Diagnosis, however, was made of retro-bulbar optic neuritis, and she was put on potassium iodide treatment. Vision continued to fail. It was suggested by one of the physicians who saw her to examine her teeth, which, being done, it was found that five of her upper teeth on the right side had been filed down level with the gums, and that upon these she was wearing a set of artificial teeth. Four days after the extraction of the stumps of teeth her vision showed an improvement. In less than two months vision was fully restored, and showed no change at a later date.

"This was undoubtedly a case of amblyopia, due to dental irritation of the fifth pair. The pathology of these cases is not definitely known, but is supposed to be due to some vaso-motor disturbances of the retina or centers of vision."

